# Editorial: Potential of natural products as drug leads possessing antioxidant and anti-aging properties

**DOI:** 10.3389/fphar.2022.1094950

**Published:** 2022-12-09

**Authors:** Fadia S. Youssef, Mohamed Fawzy Ramadan, Valentina Echeverria Moran, Adeyemi O. Aremu, Nilufar Z. Mamadalieva

**Affiliations:** ^1^ Department of Pharmacognosy, Faculty of Pharmacy, Ain-Shams University, Cairo, Egypt; ^2^ Department of Clinical Nutrition, Faculty of Applied Medical Sciences, Umm Al-Qura University, Makkah, Saudi Arabia; ^3^ Facultad de Medicina, Universidad San Sebastián, Concepción, Chile; ^4^ Research and Development Service, Bay Pines VA Healthcare System, Bay Pines, FL, United States; ^5^ School of Life Sciences, College of Agriculture, Engineering and Science, University of KwaZulu-Natal, Durban, South Africa; ^6^ Indigenous Knowledge Systems Centre, Faculty of Natural and Agricultural Sciences, North-West University, Mmabatho, South Africa; ^7^ S. Yu. Yunusov Institute of the Chemistry of Plant Substances, Academy of Sciences Republic of Uzbekistan (UzAS), Tashkent, Uzbekistan

**Keywords:** natural product, drug discovery, antioxidant, anti-aging, secondary metabolites, pharmaceutical formulation

Natural products display a wide array of medicinal values and constitute less expensive candidates with lower adverse effects and higher acceptabilities among a large number of patients worldwide compared to synthetic drugs (Thabet et al., 2018). In addition, natural products are present in food, with pharmaceutical and cosmetic industries exploiting their beneficial multipurpose effects (Al-Madhagy et al., 2019). Free radicals are considered to be among the crucial factors that exacerbate accelerated aging. Thus, a healthy, well-balanced diet and herbal product consumption are expected to slow down and even counteract the deleterious effects of free radicals (Barja, 2004). Skin aging is caused by an observed alteration in the equilibrium between dermal degenerative and regenerative mechanisms that ultimately cause epidermal thinning and wrinkling. Concomitantly, deleterious changes in the skin are associated with deterioration and loss of collagen and elastin fibers accompanied by reduced circulatory perfusion (Altyar et al., 2020). Evidence has shown that a wide array of natural-based products significantly restored this disturbed equilibrium and concomitantly stimulated the production of elastin and collagen in addition to exerting transient moisturizing effects (Park et al., 2020). Furthermore, oxidative stress plays a pivotal role in aging, based on the hypothesis that age-related functional disruptions heavily depend on the accumulation of reactive oxygen and nitrogen species (RONS), which induce damage and can be counteracted through the consumption of natural products (Salazar-García and Corona, 2021). Thus, various research articles on this Research Topic highlighted that using natural products as drug leads can combat oxidative stress and aging.

In accordance with this concept, Wu et al. investigated the role of capsaicin, Capsicum annuum L. (Solanaceae), a major secondary metabolite in chili pepper for the alleviation of ultraviolet (UV) radiation–induced skin damage. It is worth highlighting that capsaicin was previously used in a wide array of pharmaceutical applications such as anti-inflammatory, pain relief, and psoriasis treatment, in addition to the prohibition of melanogenesis induced by UV irradiation. Briefly, the exposure of cultured dermal fibroblasts to UV radiation caused a reduction in the synthesis of collagen that was accompanied by an elevation in matrix metalloproteinases (MMPs) expression, the production of reactive oxygen species (ROS), as well as enhanced Erk and c-Jun phosphorylation, resulting in skin damage. However, pre-treatment with capsaicin in a dose-dependent manner inhibited the decrease in dermal fibroblast collagen by counteracting the production of ROS *in vitro* and *in vivo* models.

Additionally, the role of oridonin, a diterpenoid obtained from *Rabdosia rubescents* Linn. (Lamiaceae), in delaying aging *via* the AKT signaling pathway was investigated by An et al. Oridonin prohibited cellular senescence in human diploid fibroblasts, as evidenced by the 59.8% reduction in senescence-related β-galactosidase staining, relative to the model group. Furthermore, the oridonin intervention group experienced a notable 48.5% reduction in the positive cell rate compared with the elderly control group. Besides, oridonin effectively elongated the yeast lifespan by 48.9%, prolonged the lifespan of naturally aged mice by 21.6%, and maintained their health status. Furthermore, oridonin hindered mouse and cellular senescence triggered by doxorubicin, with a 53.8% elongation in the mice’s average lifespan. This could be mechanistically interpreted by the AKT signaling pathway and reversing the genetic alteration triggered by doxorubicin.

The study conducted by Zhou et al. showed that morroniside, a major iridoid glycoside in *Cornus officinalis* Torr. ex Dur (Cornaceae), alleviated intervertebral disc degeneration (IVDD) which causes chronic low back pain and disability in the elderly. A lumbar spine surgery-induced instability mouse IVDD model and *in vitro* hydrogen peroxide–induced nucleus pulposus (NP) cell senescence were used to evaluate the effect of morroniside on NP cell senescence as well as on the pathogenesis of IVDD. The results of this study showed that morroniside effectively ameliorated IVDD, accompanied by a pronounced enhancement in extracellular matrix metabolism that was further confirmed by histopathological examination. It also decreased SA-β-gal activities and p53 and p21 expressions which serve as senescence indicators. Moreover, it reduced ROS-induced aberrant activation of Hippo signaling *via* prohibition of Mst1/2 and Lats1/2 phosphorylation and reversed Yap/Taz reduction. Thus, morroniside afforded a novel strategy for the alleviation of IVDD through protection against NP cell senescence and prohibition of the ROS-Hippo-p53 pathway.

Globally, the use of plants for managing skin diseases is common in various ethnic groups. Nowak et al. investigated the antioxidant, anti-inflammatory, antimicrobial, wound-healing, and anti-aging potential of *Epilobium angustifolium L.* (Onagraceae) topical hydrogels that are traditionally popular in the treatment of skin diseases. The results revealed that various hydrogels prepared from plant extracts, namely, ethanol, isopropanol, and water showed significant antioxidant potentials, as evidenced by their high phenolic acid contents determined by high-performance liquid chromatography (HPLC). Furthermore, the hydrogels elicited a notable antimicrobial activity, particularly *versus Enterococcus faecalis*, *Streptococcus pneumonia*, *Escherichia coli*, *Sarcina lutea*, *Enterococcus faecium*, and *Bacillus pseudomycoides* in contrast to *Staphylococcus aureus*, *Bacillus subtilis*, and *Streptococcus epidermidis* which were the least susceptible microbes reported. Moreover, all the tested extracts showed promising inhibition of proteases, protein denaturation, and lipoxygenase enzymes, accounting for their anti-inflammatory activities. The gels prepared from ethanol and isopropanol extracts showed pronounced wound healing activities with good skin penetration, as illustrated by *in vitro* test using Franz diffusion cells. Thus, *E. angustifolium* may afford a promising therapeutic strategy as a dermatologic and cosmetic preparation to alleviate oxidative stress and skin disorders.

Polysaccharides are recognized as functional components in plants and may exert biological effects. Zhao et al. conducted a study on two *Pogostemon cablin* Benth. (Lamiaceae) polysaccharide fractions (PCB-1 and PCB2-1) which were extracted using water and purified using Sepharose chromatography to reveal their antioxidant potentials. PCB-1 and PCB2-1 presented sugar contents of 90.23% and 88.61%, with molecular weights of 97.8 and 12.8 kDa, respectively. Analysis of the existing monosaccharides revealed the presence of rhamnose, mannose, galactose galacturonic acid, arabinose, and glucose at different molar ratios. Both polysaccharide fractions exhibited potent antioxidant activities, as evidenced by their pronounced *in vitro* scavenging behaviors *versus* hydroxyl radicals as well as their metal ion-chelating and ferric-reducing abilities. Besides, PCB-1 exhibited a notable *in vivo* antioxidant activity that was observed *via* elevated catalase, superoxide dismutase, and glutathione peroxidase levels in oxidatively damaged mice model, with a concomitant reduction of malondialdehyde observed in the liver and serum, thus serving as an antioxidant in functional foods.

Aging is known to be the main cause of muscle atrophy. As a potential therapeutic strategy, Lee et al. investigated the leaf extract of *Ricinus communis* L., castor oil, (Euphorbiaceae) for its role as an antioxidant in counteracting skeletal muscle atrophy and retaining redox homeostasis. LC-MS/MS revealed the presence of 30 compounds, with rutin constituting the major compound. Furthermore, rutin-rich *Ricinus communis* leaf extract exhibited a potent oxidative stress–combating activity *via* the Nrf2 signaling pathway, as evidenced by its ABTS and DPPH scavenging activities and its ability to reduce dexamethasone (DEX)-induced myotube atrophy and mitochondrial oxidative damage in C2C12 cells. Thus, *Ricinus communis* L. and its rutin-rich extract may serve as an excellent functional food for muscle health.

Historically, many plants have been used as food and medicine and may exert antioxidant effects to mitigate aging and age-related diseases. Li et al. assessed the antioxidant activities of three acidic polysaccharides obtained from different organs (rhizomes, roots, and aerial parts) of *Codonopsis pilosula* Franch. (Campanulaceae) by gel filtration and ion exchange chromatography. The acidic polysaccharides obtained from roots and aerial parts showed antioxidant potentials, whereas that obtained from rhizomes exhibited low activity. Furthermore, structural characterization of the bioactive fractions showed the presence of various monosaccharides in different ratios. These polysaccharides were mainly composed of 1,4-linked galacturonic acid containing long homogalacturonan regions and side chains composed of arabinogalactan type I and arabinogalactan type II. Additionally, both polysaccharides exhibited potent antioxidant activities by elevating superoxide dismutase and catalase and reducing MDA. These results were further consolidated *in vivo* using a *Caenorhabditis elegans* model, regulating nuclear localization of the DAF-16 transcription factor.

In conclusion, this Research Topic supports the fact that natural products may serve as sustainable sources of chemical entities that can act as drug leads for many pharmaceutical industries to counteract aging and oxidative stress. This is highly welcomed by many people globally. However, using naturally occurring herbal products requires many precautions during Research Topic and storage owing to the drastic effects of environmental conditions on the levels and types of existing secondary metabolites. Thus, comprehensive metabolic profiling and standardization should be performed to guarantee the activities of the tested botanical drugs. Hence, both botanical drugs and isolated compounds should be accompanied by preclinical and clinical studies to further consolidate the assumed biological activities and their suitabilities for processing as pharmaceutical dosage forms. Moreover, pharmacokinetic, pharmacodynamic, and toxicity studies should be conducted to further ascertain the efficacy and safety profiles of all examined samples.
